# Polyploidy and the relationship between leaf structure and function: implications for correlated evolution of anatomy, morphology, and physiology in *Brassica*

**DOI:** 10.1186/s12870-016-0957-3

**Published:** 2017-01-05

**Authors:** Robert L. Baker, Yulia Yarkhunova, Katherine Vidal, Brent E. Ewers, Cynthia Weinig

**Affiliations:** 1 Department of Botany, University of Wyoming, Laramie, WY 82071 USA; 2 Program in Ecology, University of Wyoming, Laramie, WY 82071 USA; 3 Department of Molecular Biology, University of Wyoming, Laramie, WY 82071 USA

**Keywords:** Brassica, Polyploidy, Phenotypic integration, Leaf morphology, Leaf anatomy, Leaf physiology, Triangle of U, Hybridization, Whole genome duplication

## Abstract

**Background:**

Polyploidy is well studied from a genetic and genomic perspective, but the morphological, anatomical, and physiological consequences of polyploidy remain relatively uncharacterized. Whether these potential changes bear on functional integration or are idiosyncratic remains an open question. Repeated allotetraploid events and multiple genomic combinations as well as overlapping targets of artificial selection make the *Brassica* triangle an excellent system for exploring variation in the connection between plant structure (anatomy and morphology) and function (physiology). We examine phenotypic integration among structural aspects of leaves including external morphology and internal anatomy with leaf-level physiology among several species of *Brassica.* We compare diploid and allotetraploid species to ascertain patterns of phenotypic correlations among structural and functional traits and test the hypothesis that allotetraploidy results in trait disintegration allowing for transgressive phenotypes and additional evolutionary and crop improvement potential.

**Results:**

Among six *Brassica* species, we found significant effects of species and ploidy level for morphological, anatomical and physiological traits. We identified three suites of intercorrelated traits in both diploid parents and allotetraploids: Morphological traits (such as leaf area and perimeter) anatomic traits (including ab- and ad- axial epidermis) and aspects of physiology. In general, there were more correlations between structural and functional traits for allotetraploid hybrids than diploid parents. Parents and hybrids did not have any significant structure-function correlations in common. Of particular note, there were no significant correlations between morphological structure and physiological function in the diploid parents. Increased phenotypic integration in the allotetraploid hybrids may be due, in part, to increased trait ranges or simply different structure-function relationships.

**Conclusions:**

Genomic and chromosomal instability in early generation allotetraploids may allow *Brassica* species to explore new trait space and potentially reach higher adaptive peaks than their progenitor species could, despite temporary fitness costs associated with unstable genomes. The trait correlations that disappear after hybridization as well as the novel trait correlations observed in allotetraploid hybrids may represent relatively evolutionarily labile associations and therefore could be ideal targets for artificial selection and crop improvement.

**Electronic supplementary material:**

The online version of this article (doi:10.1186/s12870-016-0957-3) contains supplementary material, which is available to authorized users.

## Background

Polyploid species form when unreduced gametes from one or more parent species are fertilized, resulting in an increased number of chromosomes and consequently increased genome size. Autopolyploidy occurs when the parent plants that form polyploids are from the same species, and allopolyploidy occurs when the parent plants are from different species (reviewed in [1]). In addition to genetic incompatibilities with the parent species, the increased number of chromosomes and genetic material in polyploid individuals leads to immediate changes in morphological, anatomical, and physiological characteristics relative to the parent species [2–4]. Whether these changes bear on functional integration or are idiosyncratic remains an open question. Both types of polyploidy result in sympatric speciation among plants [5, 6] and are thus important to understanding evolutionary dynamics. However, polyploidy events are often ancient and distributed across the plant phylogeny, making it difficult to draw general conclusions about the immediate effects of polyploidy [7].

In addition to speciation of wild plants, many crop species are polyploids and exhibit heterosis [7, 8], including increased growth [9], fruit size [10], drought tolerance [11], disease resistance [12], and ecological niche diversification [13]. Polyploids have been of particular interest to plant breeders in part because of the beneficial aspects of polyploidy, but also because polyploidy often leads to breakdowns in reproductive incompatibility systems and facilitates transfer of beneficial genomic regions among individuals or species [14]. The economically important genus *Brassica* has experienced multiple polyploidy events, including a polyploid event resulting in whole-genome-duplication (WGD) that occurred after divergence from the genus *Arabidopsis* [15–17]. After polyploidization, additional genetic and eipigenetic changes can cause genome shock and lead to phenotypic instability [18, 19]. Gene copies may be released from purifying selection and undergo sub-or neofunctionalization or may simply be lost [20–24]. Subsequent to allopolyploidy events, species in *Brassica* have experienced biased gene loss [25]: genes are often lost preferentially from one parent species rather than another causing polyploids to ultimately become functional diploids [26].

Within the genus *Brassica,* the classic triangle of U [27] consists of six crop species, including three functional diploid species at each point of the triangle (*B. rapa*, *B. nigra*, and *B. oleracea*). These functional diploids have hybridized to form three distinct allotetraploids (*B. carinata*, *B. juncea*, and *B. napus*), which have subsequently undergone biased gene loss and genome reorganization [21]. Within each species, repeated artificial selection has occurred for different targets of harvest: underground storage organs (turnips), leafy greens (cabbages, bok choy), axillary branches (Brussels), floral parts (cauliflower, broccolinis), or seeds (oil seeds) [28]. Within a single diploid (*B. rapa*), physiological differences are strongly associated with stomatal density, leaf anatomy, and crop type [29].

Polyploidy is well studied from a genetic and genomic perspective, but the morphological, anatomical, and physiological consequences of polyploidy remain relatively uncharacterized; datasets are necessary for a comprehensive understanding of the formation and persistence of polyploids as well as their evolutionary and agroecological implications [30]. Repeated allotetraploid events and multiple genomic combinations as well as overlapping targets of artificial selection make the *Brassica* triangle an excellent system for exploring variation in the connection between plant structures (anatomy and morphology) and function (physiology) as well as studying the effects of artificial selection and polyploidy. We use a panel of *Brassica* triangle species to ask whether repeated allopolyploid events that generated *B. carinata, B. juncea,* and *B. napus* result in altered trait expression and trait correlations. Specifically, we ask whether structural aspects of morphology and anatomy are correlated with functional physiology and test the hypothesis that allotetraploidy results in disintegration of phenotypic trait correlations allowing for transgressive phenotypes and additional evolutionary and crop improvement potential.

## Methods

### Species description

*Brassica* is a genus within the Brassicaceae that includes 19 species [31]. We focus on six annual to bi-annual species within *Brassica*. Three of these are functionally diploids (*B. nigra, n* = 8, BB*; B. oleracea, n* = 9, CC*; B.* and *B. rapa, n* = 10, AA)*.* The parent species have interbred in all possible combinations to generate three allotetraploid progeny with different genomic combinations: *B. carinata* (*n* = 17, BBCC)*, B. juncea* (*n* = 18, AABB)*,* and *B. napus* (*n* = 19, AACC) [32]. Seeds for accessions were obtained from the USDA Germplasm Information Network (GRIN)’s North Central Regional Plant Introduction Station at Ames, Iowa, USA and the Centre for Genetic Resources (CGN) at Wageningen UR, The Netherlands (Table 1). The Triangle of U is a useful conceptual model for understanding the general relationships between the six *Brassica* taxa studied here. However, the exact relationships between the diploid and polyploid accessions remains uncharacterized and the specific diploid accessions we used are unlikely to be the direct progenitors of the allotetraploid hybrids as *Brassica* allotetraploids are thought to have evolved multiple times. *B. napus* and *B. juncea* in particular likely have polyphyletic origins [33–35]. We utilized multiple accessions per taxon in part to encompass within-species phenotypic variation that may result from different polyploidy events and subsequent genetic divergence.

**Table 1:** Accession information and sample sizes for plant material used. Crop type and collection information are derived from the GRIN and CGN databases

Species	Accession ID	Number^a^	Source
*B. carinata*	CGN03952	5	CGN
	CGN03969	5	CGN
	CGN03976	5	CGN
*B. juncea*	PI 173857	5	GRIN
	PI 257240	5	GRIN
	PI 470241	5	GRIN
	PI 633094	4	GRIN
	PI 120923	5	GRIN
*B. napus*	CGN06897	4	CGN
	CGN07230	5	CGN
	CGN14113	5	CGN
	CGN17374	5	CGN
*B. nigra*	CGN06619	5	CGN
	CGN06620	5	CGN
	CGN06627	4	CGN
*B. oleracea*	CGN07129	5	CGN
	CGN14031	5	CGN
	CGN14070	5	CGN
*B. rapa*	Ames 2795	2	GRIN
	CGN06709	3	CGN
	CGN06710	2	CGN
	CGN06711	1	CGN
	CGN06813	3	CGN
	CGN07143	3	CGN
	CGN07145	3	CGN
	PI 459016	3	GRIN
	PI 459018	3	GRIN
	PI 459020	3	GRIN

^a^Actual sample sizes for individual tests are indicated by degrees of freedom and may differ for individual analyses because of failed sample processing or due to outlier removal

### Design and plant growth

We planted five separate blocks each containing five plants from five accessions for *B. oleracea, B. carinata, B. nigra, B. napus,* and *B. juncea* and five plants from 10 accessions of *B. rapa*. Plant locations were randomized within blocks. Poor germination resulted in data collected from 2 to 5 individuals from 3 (*B. oleracea, B. carinata,* and *B. nigra*), 4 (*B. napus)* 5 (*B. juncea)* or 10 (*B. rapa*) accessions of each species (Table 1). Three seeds were planted in the center of each 3.5-in. square pot filled with Sunshine Redi-Earth Professional Growing Mix (Sun Gro Horticulture, Bellevue, WA, USA) and a slow-release fertilizer (Scotts brand Osmocote Controlled Release Classic, NPK; Scotts, Marysville, OH, USA) and covered with vermiculite. Pots were randomly located on benches in a checkerboard pattern to avoid shading and watered daily to capacity. Seedlings were thinned to one plant per pot shortly after germination.

### Data collection

After the third epicotylar leaf expanded, gas exchange measurements were recorded using a Li-Cor LI-6400 XT portable infrared gas analyzer with a leaf chamber fluorometer (Open System Vers. 4.0, Li-Cor Inc., Lincoln, NE, USA). We collected physiological data from the third epicotylar leaf from 8 a.m. to 11 a.m. each morning, including three separate estimates of photosynthetic rate (*A*_*max*_), and stomatal conductance (*g*_*s*_) that were averaged for each individual, and a single measure of *F*_*o*_ *'* (minimum florescence level in the light), *F*_*v*_ *'* (variable florescence level; *F*_*m*_ *'- F*_*o*_ *')*, *F*_*m*_ *'* (maximum florescence level), and *F*_*s*_ (steady state florescence) that were used to calculate ratios of variable to maximal florescence, *F*_*v*_ *'/F*_*m*_ *'*. Measurements were taken at a photosynthetic photon flux density (PPFD) of 1500 μmol m^−2^s^−1^ (to approximate ambient greenhouse PPFD), ref [CO_2_] of 400 μmol m^−2^s^−1^, T_leaf_ = 24 °C and vapor pressure deficit based on leaf temperature (VPDL, kPa) was kept between 1.3 and 1.7 kPa [29]. Within 36 h of physiological data collection, the third epicotylar leaf was collected at the leaf base, scanned at 600 dpi using an Perfection V700 Photo scanner (Epson America, Long Beach, CA, USA), weighed, and fixed for 24 h in formalin-aceto-alcohol (FAA; 1:1:18 ratios of formaldehyde, glacial acetic acid, and ethanol by volume) and stored in 70% EtOH.

**Fig. 1:**
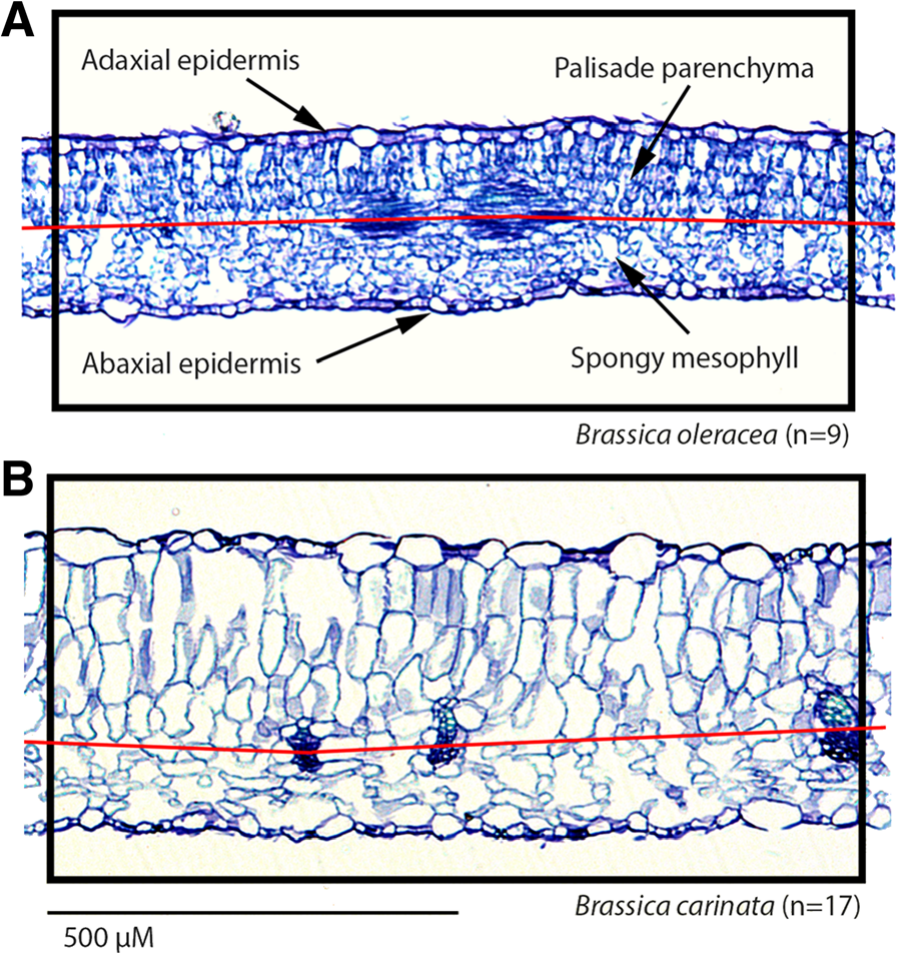
Paraffin infiltrated leaves cross-sectioned at 10 μM thickness. **a**. *Brassica oleracea* (n=9). **b**. *B. carinata* (n=17). We defined 1000 μM long sections of leaf (inside the black boxes) that avoided major veins. All measurements occur inside these boundaries. Palisade parenchyma is defined as any tissue above the mid-line of minor veins and below the adaxial epidermis. Spongy mesophyll is defined as any tissue below the mid-line of minor veins and above the abaxial epidermis. All areas refer to predefined 1000 μM long leaf sections. Scale bars are 500 µM

The fourth epicotylar leaf was collected for wet and dry mass. The remaining aboveground shoots were also collected and weighed when dry. Area and perimeter were measured on scanned leaves using ImageJ and a leaf dissection index (perimeter/area^−2^) was calculated. Leaves preserved in ethanol were dehydrated using a standard ethanol series. The ethanol was gradually replaced with Histoclear, and the leaves were infiltrated with paraffin at 60 °C [36]. Samples were embedded in paraffin, serially sectioned at 10 μM, and stained with toluidine blue O (Sakai, 1973). Sections were imaged using the 5x objective on a Zeiss Axio Lab A1 compound light microscope with a Zeiss Axiocam 105 color camera (Carl Zeiss GmbH, Jena, Germany). Tiff images were rotated in Adobe Photoshop CS6 (Adobe Systems Inc., San Jose, CA, USA), and a section of leaf 1000 μM long that avoided major veins was defined as the measurement area. Within this area, palisade parenchyma was defined as the area above the mid-point of minor veins and below the adaxial epidermis. The spongy mesophyll was defined as the area below the mid-point of minor veins and above the abaxial epidermis (Fig. 1). All anatomic measurements were collected using ImageJ [37]. Raw data are available in Additional file 1: Table S1.

### Data analysis

As a conservative approach to ensure that significant results do not derive from the effects of one or two data points, all data were subjected to an outlier analysis. Data points for each plant and trait outside three standard deviations of the grand mean (calculated including all individuals from all species) were excluded. Subsequent visual inspection of histograms and quantile-quantile plots indicated that excluding two data points for *F*_*o*_ *'* and three for *F*_*m*_ *'* and *F*_*s*_ improved normality. Significant effects of species and ploidy (*2n* vs. *4n*) were assessed using a series of one-way ANOVAs with planned contrasts for individual species effects and the effect of ploidy level (i.e. parent vs. hybrid species) in the R statistical environment (v3.2.3, [38]). We dropped all traits that lacked significant species or parent-hybrid effects from further analyses (except the principle components analysis, below). We used log-transformed data from individual plants to calculate phenotypic correlations for all six species, parents, and hybrids while using a Bonferroni correction for multiple tests (*cor.test*). We interpret correlations that are observed only in the parents as evidence that the history of selection has resulted in functional integration at the phenotypic level and that hybridization has broken down these trait correlations. We interpret correlations only observed in the hybrids as evidence that novel allelic combinations arising from allopolyploidy has the potential to generate new phenotypic correlations not observed in parent species.

To examine the basis of trait correlations (or lack thereof), we first compared trait ranges between parents and hybrids to determine whether transgressive trait values (in the hybrids) could be driving trait correlations. Increases in the trait value ranges were considered to be biologically meaningful if the allotetraploid range was 110% of the diploid parent range of values; likewise, allotetraploid trait value ranges were considered to have contracted if they were less than 90% of the diploid parent range. Increases in maximum trait values from parental to polyploidy hybrid species were considered biologically meaningful if the hybrid maximum trait value exceeded the maximum parental trait value plus 10% of the parental trait range. Decreases in the maximum trait values were considered biologically meaningful if the maximum hybrid trait value was less than the maximum parental trait value minus 10% of the parental trait range. Similarly, changes in minimum trait values were considered biologically meaningful if the minimum hybrid value was less than the minimum parental trait value minus 10% of the parental range or larger than the parental trait value plus 10% of the parental range.

The relationship between some trait pairs in the parents appeared to have three clusters of data points. We used a K-means clustering analysis with the a priori number of clusters set to the number of species (three) to ask whether trait values clustered by species in both parents and hybrids [39]. We performed a principle components analysis (using the *prcomp* function in R) to determine whether suites of structural (morphological or anatomical) or functional (physiological) traits, or a mixture, explained the main axes of variation present in the data. We use the R package *ggbiplot* to draw ellipses with normal probability contours set to 68% to help visualized the relationship between ploidy level or species [40].

## Results

### Effects of species and ploidy on individual traits

Following one-way analysis of variance tests, we performed planned contrasts to test whether there were ploidy level (parent vs. hybrid) effects. Among physiological traits, there were significant differences among individual species for *F*_*v*_ *'/F*_*m*_ *'*, *F*_*v*_', and *F*_*o*_ *'* but notably not photosynthetic capacity (*A*_*max*_), stomatal conductance (*g*_*s*_) or intrinsic Water Use Efficiency (WUE; Table 2). For physiological traits, there were no statistically significant effects of ploidy level, however there were marginally significant (*F* = 2.856 (1,91), *p* < 0.1) effects of ploidy level for *F*_*s*_. Among leaf morphological characters, there were significant parent-hybrid effects on leaf dry weight, leaf area, and leaf perimeter as well as marginally significant effects of leaf dissection index (Table 2). There were species-specific effects for Specific Leaf Area (SLA), dissection index, and leaf perimeter with marginally significant effects of leaf area. For morphological traits, there were significant parent-hybrid effects of palisade parenchyma area, adaxial epidermis area, and abaxial epidermis area with marginally significant effects (*p* < 0.1) for the ratio of palisade parenchyma to spongy mesophyll areas. There were significant species-specific effects of palisade parenchyma area and the ratio of palisade parenchyma to spongy mesophyll area and marginally significant effects of species on spongy mesophyll area (Table 2).

**Table 2:** One way ANOVA and planned contrasts for anatomical, morphological, and physiological traits

Trait	Species effect F (DF_numerator_, DF_denominator_)	Parent-hybrid effect F (DF_numerator_, DF_denominator_)
Palisade area	3.811 (5,77)**	15.614 (1,77)***
Spongy area	***2.289 (5,76).***	0.376 (1,76) NS
Palisade/spongy	3.13 (5,76)*	***3.58 (1,76).***
Palisade layers	1.467 (5,77) NS	0.034 (1,77) NS
Adaxial area	1.814 (5,76) NS	4.568 (1,76)*
Abaxial area	1.801 (5,76) NS	5.241 (1,76)*
Leaf 4 dry weight (mg)	1.696 (5,103) NS	6.396 (1,103)*
Dry shoots (g)	1.259 (5,101) NS	1.215 (1,101) NS
SLA	7.684 (5,102)***	0.168 (1,102) NS
Dissection index	6.11 (5,104)***	***3.56 (1,104).***
Area (cm^2)	***2.063 (1,104).***	5.500 (1,104)*
Perimeter (cm)	3.112 (5,104)*	11.967 (1,104)***
WUE	0.317 (5,94) NS	0.202 (1,94) NS
*A*_*max*_	1.169 (5,95) NS	2.569 (1,95) NS
*g*_*s*_	0.178 (5,95) NS	0.669 (1,95) NS
*F*_*v*_ *'*/*F*_*m*_ *'*	5.877 (5,86)***	0.241 (1,86) NS
*F*_*v*_ *'*	5.546 (5,97)***	0.003 (1,97) NS
*F*_*m*_ *'*	1.649 (5,91) NS	2.264 (1,91) NS
*F*_*o*_ *'*	4.988 (5,88)***	0.019 (1,88) NS
*F*_*s*_	1.711 (5,91) NS	***2.856 (1,91).***

Significance: 0 ‘***’; 0.001 ‘**’; 0.01 ‘*’; 0.05 ‘.’

*NS* Not Significant

### Phenotypic correlations at different ploidy levels

**Fig. 2:**
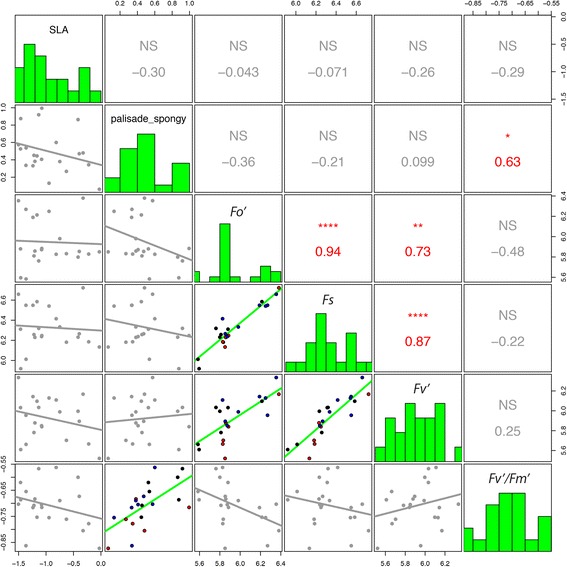
Phenotypic correlations among individual plants from the parent (2n) species. *B. rapa* (*blue*)*, B. oleracea* (*red*)*, and B. nigra* (*black*)*.* Histograms show trait distributions and correlations. Non-significant correlations are in *gray*. Palisade_spongy, the ratio of palisade parenchyma to spongy mesophyll; *p* < 0.05, *; *p* < 0.01, **; *p* < 0.001, ***, *p* < 0.0001 ****

As expected, we often observed strong correlations among leaf morphological traits (especially when considering all six species; Figure S1). For instance, leaf area was always significantly correlated with leaf perimeter (Figures S1–S3). There were significant correlations among some anatomical traits such as spongy mesophyll area and the ratio of palisade parenchyma to spongy mesophyll area, a correlation that was always significantly negative (Figures S1–S3). We also observed strong significant correlations among leaf-level physiology traits such as *F*_*o*_ *'*, *F*_*v*_ *'* and *F*_*s*_, (Figs. 1, 2, and S1–S3).

**Fig. 3:**
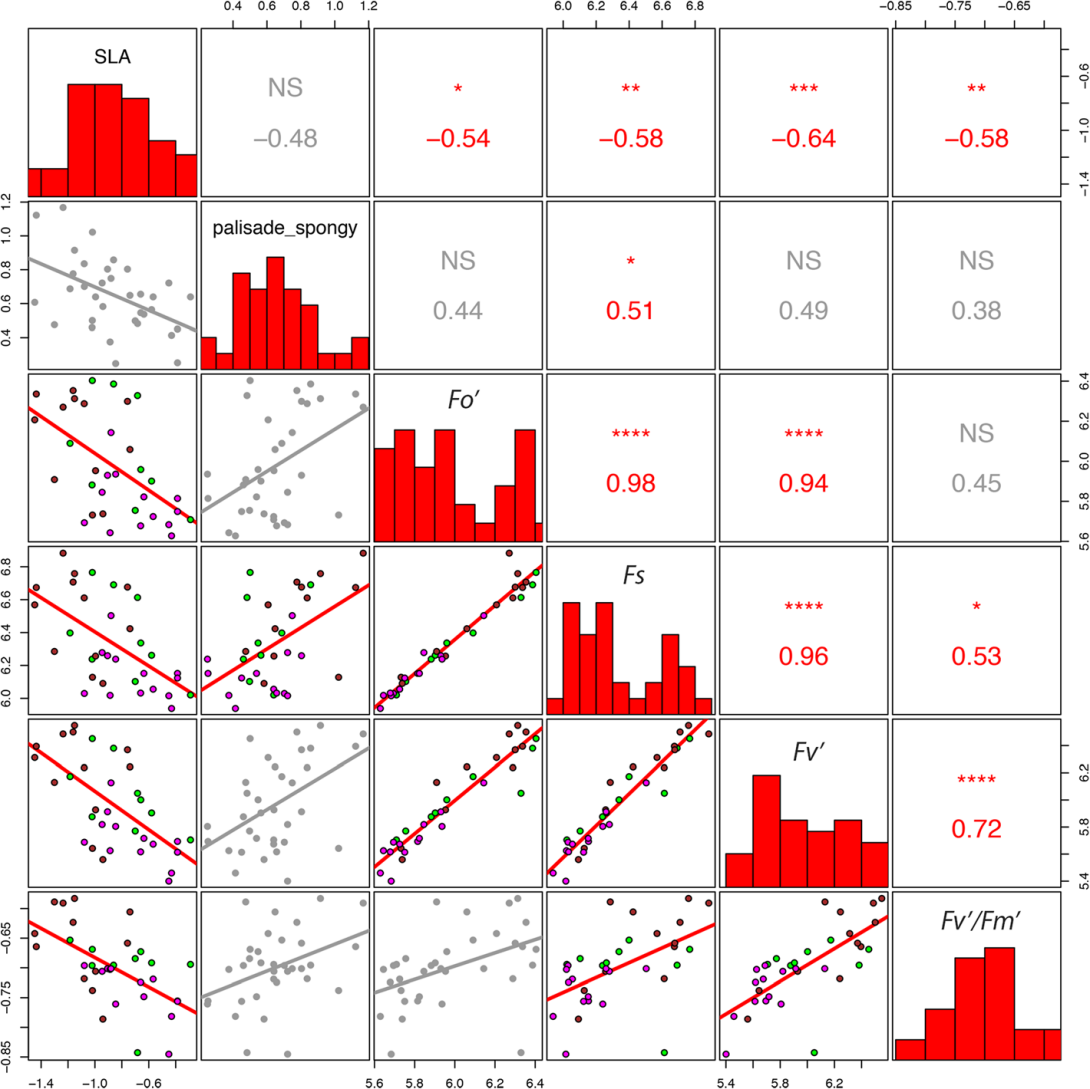
Phenotypic correlations among individual plants from the allotetraploid (4n) hybrids. *B. oleracea* (*magenta*)*, B. juncea* (*brown*)*,* and *B. carinata* (*green*). Palisade_spongy, the ratio of palisade parenchyma to spongy mesophyll; *p* < 0.05, *; *p* < 0.01, **; *p* < 0.001, ***; *p* < 0.0001, ****

We explored the relationship between leaf morphological or anatomical structure and leaf-level physiological function. Among accessions within parent (*2n*) species (*B. rapa, B. napus,* and *B. nigra*), there was a significant relationship between structure (morphologic or anatomic traits) and function (physiological traits): the ratio of palisade parenchyma to spongy mesophyll area was significantly positively correlated with *F*_*v*_ *'/F*_*m*_ *'* (*r* = 0.59, *p* < 0.05, Fig. 2). In the allopolyploid (*4n*) hybrids (*B. oleracea, B. juncea, and B. carinata*), the ratio of palisade parenchyma to spongy mesophyll area was positively correlated with *F*_*v*_ *'* and *F*_*s*_ but not *F*_*v*_ *'/F*_*m*_ *'* (Fig. 3). In the parent species, none of the bivariate correlations with SLA were significant (Fig. 2). In the allopolyploid hybrid species, SLA was significantly negatively correlated with *F*_*o*_ *'*, *F*_*v*_ *'*, *F*_*s*_, and *F*_*v*_ *'/F*_*m*_ *'* (Fig. 3). In general, we observed more phenotypic trait correlations among hybrids (18) compared to parents (8); 6 trait correlations were shared among parents and hybrids, 12 trait correlation were unique to hybrids, and only two trait correlations were unique to parents.

### Trait ranges

Because the range of trait values can affect the likelihood of detecting bivariate correlations, we compared the ranges of trait values for parent and allotetraploid species (Additional file 2: Table S2). Of the 14 traits examined, 11 traits exhibited ranges that were 10% wider for allotetraploids hybrids compared to diploid parents. Two traits (SLA and *F*_*v*_ *'/F*_*m*_ *'*) had allotetraploid trait ranges that were 10% narrower than trait ranges for diploid parents, and one trait range (*F*_*o*_ *'*) did not differ among parents and hybrids. Three traits had a hybrid minimum value that was less than the parental minimum (leaf area, dissection index, and abaxial epidermal area), and three traits had hybrid minimum values that were larger than the allotetraploid parent (spongy parenchyma area, adaxial epidermal area, and the ratio of palisade parenchyma to spongy mesophyll). Most of the expansion in trait ranges among hybrids was due to increased maximum values. Ten traits had maximum values in the allotetraploids that were larger than the parental hybrid maximum values, and one trait (SLA) had a hybrid maximum value that was less than the parental maximum value. Taken together, 11 traits were transgressive (smaller minimums, larger maximums, or larger ranges) in the allotetraploids compared to the diploid parents.

**Fig. 4:**
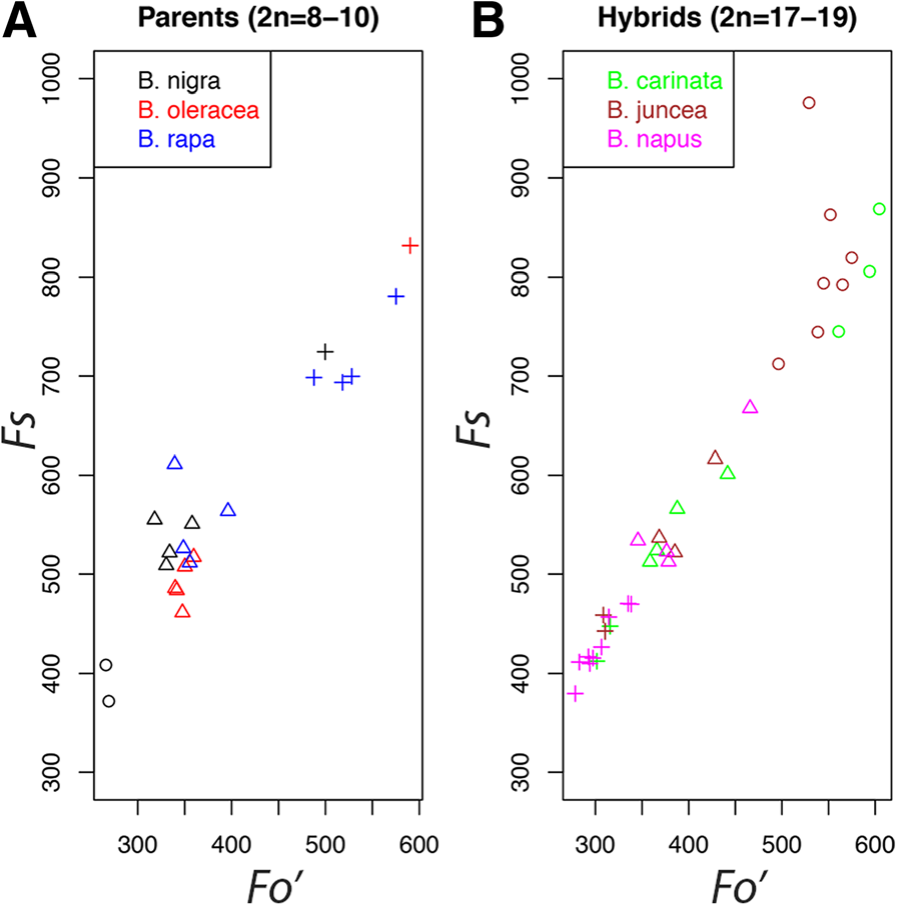
Cluster analysis of the bivariate relationship between Fo and Fs for diploid parent and allotetraploid hybrid species indicates that the three apparent clusterings of data (cluster 1, *circles*; cluster 2, *crosses*; and cluster 3, *triangles*) is not attributable to individual species (designated by *color*). Each data point represents an individual plant

K*-means clustering*: The correlations for *F*_*o*_ *'* and *F*_*s*_ *'* trait values appeared to fall into three distinct clusters of data in the diploid parents. We tested whether each of the three species fell into an individual cluster using a k-means clustering analysis with the a priori number of clusters set to three. In the parents, although the cluster analysis found three distinct clusters of data (represented by three different shapes), all three species were found in two of the three clusters (Fig. 4a) and the third cluster consisted solely of *B. nigra* individuals. Because we set the a priori number of clusters to three, we also identified three clusters in the relationship between *F*_*o*_ *'* and *F*_*s*_ for the allotetraploid hybrids. However, these clusters were less distinct, and two of the clusters consisted of individuals from all three allotetraploid hybrid species while the third cluster consisted of individuals from *B. juncea* and *B. carinata* (Fig. 4b). Even in cases when there appeared to be distinct clusters of data in bivariate correlations, these clusters were not attributable to individual species.

### Principle component analysis

**Fig. 5:**
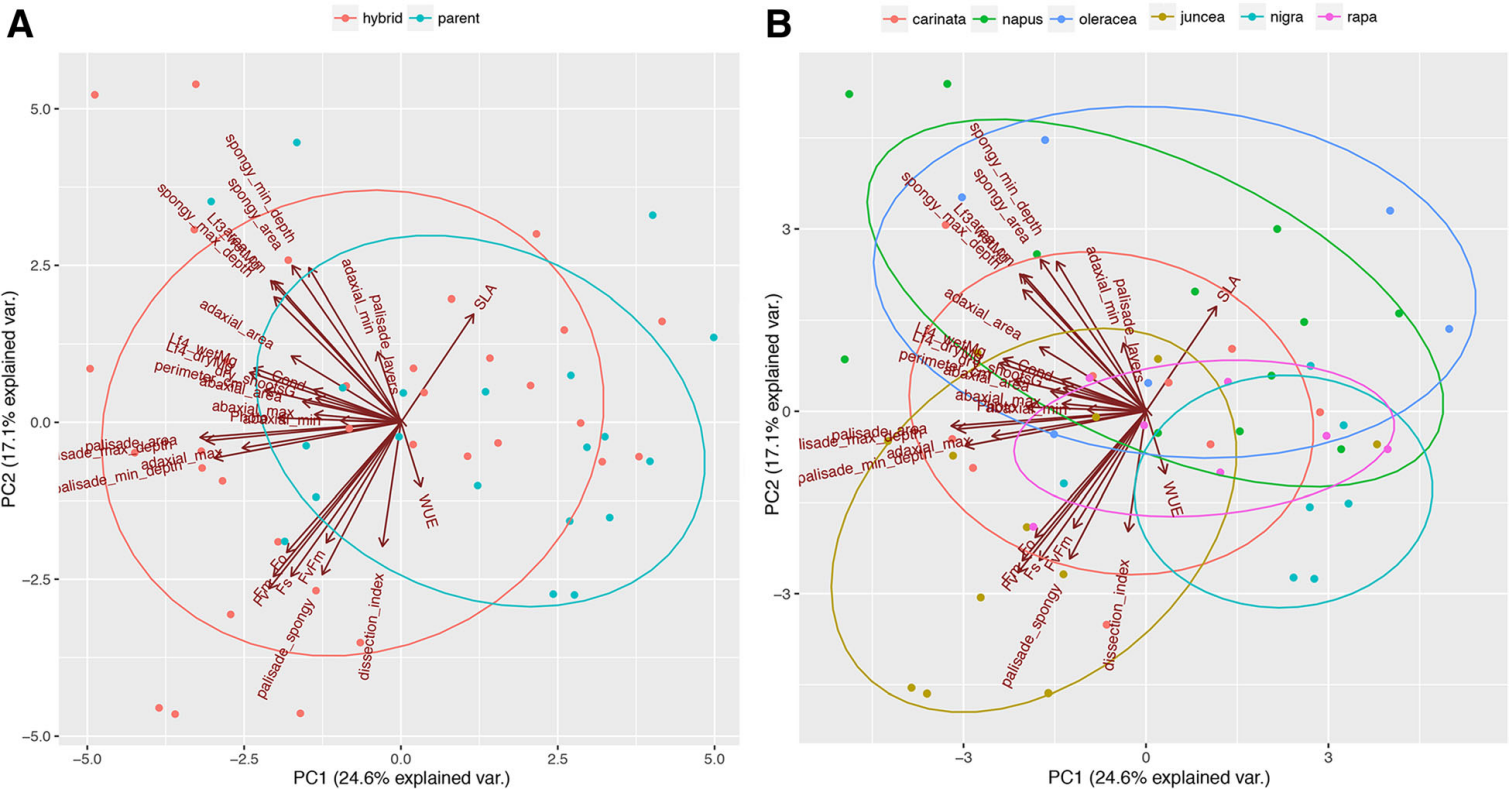
The first two axes of the PCA, which explain 24.6 and 17.1% of the variation in the data, respectively. Diploid parents and allotetraploid hybrids are largely overlapping, and the distinction between them can be attributed to SLA and WUE (5A). Individual species are also largely overlapping (5B), however the transgressive nature of the allotetraploid hybrids is evident as two of the diploid parents (*B. rapa, B. oleracea,*) occupy the center of variation whereas two of the allotetraploid hybrids (*B. juncea, B. napus*) explore the extremes of the variation evident in the data*. Lf4_wetMg,* the wet mass of the 4th leaf in mg; *dry_shootsG,* the mass of dried shoots in grams; *Lf4_dryMg*, the dry mass of the 4th leaf in mg; *area_cm*, the area of the 4th leaf in cm^2^; perimeter_cm, the perimeter of the 4th leaf in cm; *dissection_index*; the dissection index of the 4th leaf; *SLA*, specific leaf area of the 4th leaf; *palisade_layers*, the number of vertical layers of cells in the palisade parenchyma, *palisade_max_depth,* the maximum depth of palisade parenchyma in μM, *palisade_min_depth*, the minimum depth of the palisade parenchyma in μM; *palisade_area*, the area of palisade parenchyma in a 1000 μM long section of leaf in μM; *spongy_max_depth,* the maximum depth of spongy mesophyll in μM; *spongy_min_depth*, the minimum depth of spongy mesophyll in μM; *spongy_area*, the area of spongy mesophyll in a 1000 μM long section of leaf in μM, *adaxial_max,* the maximum depth of adaxial epidermis in μM, *adaxial_min*, the minimum depth of the adaxial epidermis in μM, *adaxial_area*, the area of adaxial epidermis in a 1000 μM long section of leaf in μM, *abaxial_max*, the maximum depth of the abaxial epidermis in μM; *abaxial_min*, the minimum depth of abaxial epidermis, *abaxial_area*, the area of the abaxial epidermis in a 1000 μM long section of leaf; *palisade_spongy,* the ratio of palisade parenchyma area to spongy mesophyll area; *Photo*, photosynthetic capacity (*A*_*max*_); *Cond*, stomatal conductance (*g*_*s*_); *WUE*, water use efficiency

We used a Principle Components Analysis (PCA) to examine the main sources of variation in the data. Taken as a whole, the first axis of variation explains 24.5% of the variation in the data, the second axis explains 17.1% of the variation, and the third axis explains 10.5% of the variation. All subsequent axes explain less than 10% of the variation. SLA and WUE load positively onto the first axis of variation whereas all other traits load negatively (Fig. 5). The second axis of variation has most aspects of physiology (except photosynthetic capacity and stomatal conductance) loading negatively while most aspects of morphology (except dissection index) load positively. Five of the fourteen anatomical traits load negatively. On the third PCA axis, while most morphological traits load negatively (except dissection index and SLA), most anatomical traits load positively (except spongy mesophyll maximum and minimum depths) and most physiological traits load negatively (except photosynthetic capacity and stomatal conductance; Additional file 3: Table S3).

Color coded data and normalized ellipses demonstrate that for the first PCA axes, parents and hybrids have a large degree of overlap (Fig. 5a), however the differences that do exist between parents and hybrids can be largely attributed to SLA and WUE (Fig. 5a). Individual species also explain large amounts and types of variation. However, the transgressive nature of the allotetraploids is evident as the two of the diploid parents (*B. rapa*, *B. nigra*,) cluster towards the middle while the two of the allotetraploids (*B. juncea*, *B. napus*) account for much of the extremes in the data variation (Fig. 5b).

## Discussion

Physiological function such as light gathering and harvest are critical to plant survival and reproduction. Many of these processes occur within leaves, where the ability to maintain hydraulics, conduct gas exchange, regulate temperature, harvest light, and dissipate excess light energy are dependent on leaf anatomic and morphological structures. Given the complex and multifaceted roles leaves play, one expectation is that leaf morphological and anatomical structures should be highly integrated with leaf-level physiological function. One of the most obvious examples of leaf structure-function relationship is kranz anatomy in C4 plants, where specialized bundle sheath and mesophyll cells surround vascular bundles and provide improved separation and improved efficiency of carboxylation and decarboxylation reactions [41]. Leaf-level structure-function relationships are also evident in larger datasets that include both C3 and C4 plants [42]. However, studies of the relationship between leaf structure and function often examine only morphological aspects of leaves, such as specific leaf area or leaf thickness, rather than internal anatomical variation among leaves (e.g. [42]). From a functional perspective, internal anatomy may be much more directly related to physiological processes, including gas exchange and carbon assimilation rates than external morphology [43]. Further, evolutionary diversification and crop improvement can be constrained by trait correlations [44, 45]. Genomic doubling, specifically allopolyploid formation may break down phenotypic trait correlations, leading to phenotypic instability and opening up new potential targets for natural and artificial selection [4, 46]. We examine phenotypic trait correlations among structural aspects of leaves including external morphology and internal anatomy with leaf-level physiology among several species of *Brassica.* We compare diploid parental species with allotetraploid hybrids and ascertain that patterns of phenotypic integration among structural and functional traits change after large-scale genomic reorganization such as the occurrence of polyploidy.

Within the genus *Brassica*, a cluster of six closely related species make up the classical “Triangle of U” [27]. Three diploid species represent the triangle’s vertices, each with its own alphabetic genomic designation: *Brassica rapa* (*n* = 10, AA genome), *B. oleracea* (*n* = 8, CC genome), and *B. nigra* (*n* = 8, BB genome). The three diploids have crossed in every possible combination to generate three allotetraploid hybrids, located on the edges of the triangle: *B. juncea* (*n* = 18, AABB), *B. napus* (*n* = 19, AACC), and *B. carinata* (*n* = 17, BBCC) [47–49]. Within each species, there has been considerable artificial selection for multiple, disparate crop varieties that can be generally partitioned into three morpho-types: those with root-like underground storage structures, leafy-green vegetables, and high oil content seed producers. Within the diploid species *Brassica rapa*, crop types and experimental populations exhibit correlations between leaf morphology, anatomy, and leaf-level physiological traits such as stomatal conductance, photosynthetic capacity, and water use efficiency [29, 50]. However, the fate of these associations after hybridization events that result in allotetraploidy remains untested. We expanded our previous *B. rapa* dataset to include multiple accessions from each of the six *Brassica* Triangle species to test the hypothesis that allotetraploidy results in trait disintegration allowing for transgressive phenotypes and additional evolutionary and crop improvement potential.

Among *Brassica* triangle species, we found significant effects of species for morphological (e.g. specific leaf area and leaf dissection index), anatomical (e.g. palisade parenchyma area, spongy mesophyll area) and physiological (e.g. *F*_*v*_ *'/F*_*m*_ *'* and *F*_*o*_ *'*) traits (Table 2). We also found significant effects of ploidy level, sometimes for traits affected by species, and sometimes independently of species-level effects. For instance, ploidy level had significant effects on leaf dry mass, and ab- and ad-axial epidermis area and marginally significant effects on *F*_*s*_ (Table 2). These results indicate that ploidy level can affect both leaf morphological and anatomic structures as well as physiological function. Our results are congruent with studies of newly re-synthesized allotetraploid *B. napus,* which demonstrate 70% of life history traits differ from the parental diploids [51], and which indicate that aspects of leaf morphology can exhibit transgressive phenotypes [52, 53].

Suites of traits governing similar aspects of organismal biology are often highly integrated. In rice, structural aspects of leaves such as leaf thickness and mesophyll cell surface area are highly inter-correlated while functional aspects of leaves such as photosynthetic rate and stomatal conductance are also highly inter-correlated [54]. However, phenotypic integration is not a forgone conclusion; recent work in tomato reveals relatively weak coordination between leaf structure and function [55]. We identified three intercorrelated suites of traits in both diploid parents and allotetraploids. Morphological traits such as leaf areas and perimeters were significantly correlated (Figures S1-S3). Anatomic traits were also correlated such as ab- and ad- axial epidermis areas (Figures S1-S3). Finally, aspects of physiology were correlated including *F*_*o*_ *'*, *F*_*v*_ *'*, and *Fs* (Figures S1-S3), likely because these values are all mechanistically related to photosystem II function. However, correlations between suites of structural traits (morphological and anatomical) were also present. For instance, SLA was correlated with the ratio of palisade parenchyma to spongy mesophyll (Figure S3). Palisade parenchyma typically consists of densely packed cells compared to the relatively large extracellular air spaces in spongy mesophyll, explaining why leaves with a high SLA would also have a higher ratio of palisade parenchyma to spongy mesophyll. Leaf area and perimeter were correlated with spongy mesophyll area (Figures S1 and S2). Spongy mesophyll tissue, which has large extracellular spaces may be less costly to construct than more densely packed palisade parenchyma and so scales more directly with leaf area, particularly for larger leaves [56].

We expected fewer structure-function relationships within allotetraploid hybrids compared to diploid parents because we hypothesized that genomic doubling would reduce the selective pressure on additional gene copies allowing them to independently sub- or neo-functionalize, resulting in a break down of trait correlations [57]. Of particular note in our study, there were no significant correlations between morphological structure and physiological function in the diploid parents. Hybridization, however, may introduce novel genomic interactions, resulting in new, transgressive phenotypes. Recent allotetraploids, including re-synthesized *Brassica* allotetraploids do often exhibit transgressive phenotypes and decreased trait correlations [58–60], potentially allowing them to explore new evolutionary space.

The allotetraploid accessions in our study, however, were not recently resynthesized, and we found more correlations between structural and functional traits for allotetraploid hybrids than diploid parents. Parents and hybrids did not have any significant structure-function correlations in common (Figs. 1, 2, S2, and S3). These results are broadly congruent with previous studies. For instance, polyploidy can contribute to disassociation of phenotypic traits and allow lineages to overcome constraints imposed by trait integration in *Dianthus broteri* [57]. During the first several generations after formation of allotetraploid *B. napus,* there can be extensive chromosomal instability, aneuploidy, and homoeologous shuffling resulting in numerous novel phenotypes with reduced viability and seed set [61]. Analyses of older, naturally occurring *B. napus* report much more stable karyotypes [62, 63]. Studies in *Brassica* have also found a larger degree of morphological and life history trait integration among established allotetraploids compared to diploid parental species [64].

Considered from the broadest functional perspective, the range of values we observed for *F*_*v*_ *'/F*_*m*_ *'* in a single genus and constant environment (0.37–0.57) fall well within the range observed at a continental scale and across multiple vegetation types (0.14–0.89) [65]. More narrowly, compared to diploid parents species, the allotetraploids we examined had increased trait ranges that were largely caused by increased maximum trait values, despite relatively low rates of photosystem II gene retention following polyploidy in *Glycine*, *Medicago*, and *Arabidopsis* [66]. The increased number of significant correlations in the allotetraploid hybrids may be due, in part, to increased trait ranges or simply different structure-function relationships. Additionally, PCA analyses identified allotetraploid species as tending to explain the extreme values within our data as compared to the diploid parental species. Our allotetraploid accessions may have already undergone a period of intense chromosomal instability and concomitant phenotypic trait disassociation that exposed novel phenotypes to natural or artificial selection and ultimately lead to genomic stability and novel phenotypic trait variances and covariances (reviewed in [67]).

## Conclusion

We examined multiple accessions from each of three allotetraploids and their functionally diploid parent species in the classical *Brassica* Triangle of U to test if leaf structure-function relationships, many of which are highly conserved across the leaves of seed plants, can change after hybridization. Novel genomic combinations and interactions allow for the break down of ancestral phenotypic trait correlations and the generation of novel trait correlations not exhibited by the parent species. Genomic and chromosomal instability in early generation allotetraploids may allow these species to explore new trait space and potentially reach higher adaptive peaks than their progenitor species could, despite temporary fitness costs [68]. The trait correlations that disappear after hybridization as well as the novel trait correlations observed in allotetraploid *Brassica* hybrids may represent relatively evolutionarily labile associations and therefore could be ideal targets for artificial selection and crop improvement.

## Additional files

Additional file 1: Table S1. Raw phenotypic data. (CSV 29 kb)
Additional file 2: Tables S2. Difference in trait ranges between parents and hybrids. (XLSX 52 kb)
Additional file 3: Tables S3. PCA loadings for the first three axes for each trait (CSV 1 kb)

## Abbreviations

CGN: Center for Genetic Resources
FAA: Formalyne acetic acid
GRIN: Germplasm Information Network
PCA: Principle component analysis
SLA: Specific leaf area
USDA: United States Department of Agriculture
VPDL: Vapor Pressure Deficit
WUE: Water Use Efficiency
